# Psychological distress among students in Egypt and Jordan during the initial months of the Gaza war

**DOI:** 10.1186/s40359-024-02161-2

**Published:** 2024-11-20

**Authors:** Mohamed Hendawy, Mohamed Abouzid, Aliaa Gamal, Aseel Ghanayem, Muna Amer, Mohammad Tanashat, Nael Kamel Eltewacy, Mohamed Mustafa Mohamed, Eman Ayman Nada, Ismail A. Ibrahim

**Affiliations:** 1 Faculty of Pharmacy, Alexandria University, Alexandria, Egypt; 2 Department of Physical Pharmacy and Pharmacokinetics, Faculty of Pharmacy, Poznan University of Medical Sciences, Rokietnicka 3 St, 60-806 Poznan, Poland; 3 Doctoral School, Poznan University of Medical Sciences, 60-812 Poznan, Poland; 4 Clinical Pharmacy Department, Faculty of Pharmacy, Deraya University, Minia, Egypt; 5 Eltewacy Arab Research Group, Cairo, Egypt; 6 School of Medicine, The University of Jordan, Amman, 11942 Jordan; 7 Faculty of Medicine, Jordan University of Science and Technology, Irbid, Jordan; 8 Faculty of Medicine, Yarmouk University, Irbid, Jordan; 9 Faculty of Pharmacy, Beni-Suef University, Beni-Suef, Egypt; 10 Faculty of Pharmacy, Deraya University, New Minya, Minya, Egypt; 11 Faculty of Pharmacy, Tanta University, Tanta, Egypt; 12 Faculty of Health Sciences, Fenerbahce University, Istanbul, Turkey

**Keywords:** War, Anxiety, Depression, Stress, Healthcare students, Psychache, COVID-19, Pandemic

## Abstract

**Background:**

Psychological distress has significantly impacted students in Egypt and Jordan. These countries have faced many challenges, including the COVID-19 pandemic, the fallout from the conflict in Syria, and the war in Ukraine. These crises have had far-reaching consequences, affecting the economy, food security, and energy supplies, particularly with the increased number of refugees in these countries. Amid these existing complexities, the ongoing war in Gaza further exacerbates the situation, compounding mental health challenges. Therefore, this study aimed to analyze how the war in Gaza impacted the mental health of students in Egypt and Jordan.

**Methods:**

We conducted a cross-sectional study involving students from Egypt and Jordan between December 2023 and January 2024. The questionnaire incorporated the Brief Symptom Inventory 18 (BSI-18) to assess the overall general distress score, as well as the domains of anxiety, somatization, and depression.

**Results:**

A total of 1509 Jordanian and Egyptian students were included in the study, of which 66% were female. Approximately 18% of the students had an elevation (≥ 50) in their BSI-18 total score. Females scored significantly higher in general distress [30 (18–44.25) vs. 24 (11–41), *p* < 0.001], and all three dimensions. The univariate predictors for elevated general distress (≥ 50) were being Egyptian (OR 1.49, 95% CI 1.08 to 2.08, *p* = 0.014), watching social media multiple times a day (OR 3.04, 95% CI 1.97 to 4.69, *p* < 0.001), and having a Palestinian connection (friend, neighbor, colleague, or relative) (OR 1.63, 95% CI 1.23 to 2.16, *p* < 0.001). These three predictors were retained in the backward stepwise multivariate regression analysis model. The univariate predictors for moderate and marked general distress (≥ 57) were watching social media multiple times a day (OR 3.26, 95% CI 1.78 to 5.99, *p* < 0.001) and having a Palestinian connection (OR 1.49, 95% CI 1.05 to 2.14, *p* = 0.026). Only the former was retained in the backward stepwise multivariate regression analysis.

**Conclusion:**

General distress has increased among students in Egypt and Jordan throughout the War in Gaza. Mental, psychological, and social support activities are necessary for these students, particularly those watching social media multiple times daily. The development of coping skills to manage the additional stressors of war and socioeconomic status necessitates further research within this group of students.

**Supplementary Information:**

The online version contains supplementary material available at 10.1186/s40359-024-02161-2.

## Introduction

On October 7th, 2023, Hamas, a Palestinian armed group, initiated Operation Al-Aqsa Flood inside Israel, resulting in the loss of 1,200 lives [1] and the capture of 253 hostages [2]. In response, Israel formally declared war against Hamas [3], and the Israeli military conducted air strikes on Gaza and launched a ground offensive. By December 20, 2023, over 20,000 people have reportedly died in Gaza, according to the Hamas-run government. The rate of casualties in this conflict has been noted as particularly high, as observed by Professor Michael Spagat, an expert in examining death tolls in global conflicts [1].

According to the UNICEF Middle East and North Africa Humanitarian Situation Report [4], the Middle East and North Africa region (MENA) has already faced numerous challenges. These included 5,662,199 new cases of COVID-19 with 30,726 associated deaths in 2022, the multifaceted crisis in Syria [5], and the war in Ukraine in 2022. These events have impacted the region regarding political negotiations, military actions, humanitarian aid, food security, and gas and oil supplies [4]. It was highlighted that Egypt’s situation is particularly alarming considering its role as both a transit and destination country for migrants, refugees, and asylum seekers. Egypt is home to nine million such individuals, with an estimated one million classified as vulnerable [4].

Moreover, Jordan is hosting the world's second-highest share of refugees per capita. More than 760,000 refugees are registered with UNHCR, predominantly from Syria, with large groups also from Iraq, Yemen, Sudan, and Somalia [6]. Therefore, the ongoing war in Gaza further complicates these issues, especially when the two countries share borders with Palestine.

Psychological distress, characterized by non-specific symptoms of stress, anxiety, and depression [7], has significantly affected students in Egypt and Jordan due to the COVID-19 pandemic [8–10]. This distress can negatively impact various aspects of an individual’s life, including their studies, relationships, sleep, and more [11]. The prevalence of psychological distress varies across countries, influenced by the robustness of their healthcare systems and the restrictions imposed due to the pandemic [12].

In addition to the pandemic, war has also been reported to exacerbate students’ psychological distress. For instance, a study involving 2,430 Libyan students during a civil war revealed that 64.5% of the participants experienced varying degrees of anxiety related to displacement from their homes, as measured by the General Anxiety Disorder-7 scale [13]. Similarly, a study of 350 Syrian students showed a prevalence of 60.6% for depression, 35.1% for anxiety, and 52.6% for stress, as measured by the depression, anxiety, and stress scale [14]. Another study conducted on 1,369 students in conflict-ridden Syria found that 53% suffered from post-traumatic stress disorder, and 62% struggled with problematic anger issues [15].

Therefore, this study aimed to analyze how the war in Gaza impacted the mental health of students in Jordan and Egypt. The goal is to provide a suggested guideline for supportive resources at academic institutions for students with mental health challenges.

This study will address the following research questions:

- What is the impact of demographic parameters (gender, frequency of watching social media posts about war, acquaintance with a Palestinian person, nationality) on the levels of anxiety, somatization, and depression among students?
- What are the correlations between age and the levels of anxiety, somatization, and depression among students?
- What are the predictors for general distress?

## Methods

### Study Design

The study was conducted using a cross-sectional design, adhering to the Strengthening the Reporting of Observational Studies in Epidemiology (STROBE) guidelines, which ensure transparency and reproducibility in observational research [16]. Specifically, details on how these guidelines were implemented are provided in Supplementary File 1. Adherence to STROBE helped ensure a systematic approach to the study design, participant selection, data collection methods, and statistical analyses, allowing for clarity and replicability. We used an anonymous, self-administered online survey tool through the "Microsoft Forms" platform.

### Study population

The inclusion criteria for individuals who consented to participate in the study included age ≥ 18 years and being a student and a citizen of Egypt or Jordan. There were no restrictions on gender or socioeconomic level. The exclusion criteria were all participants less than 18 years old who refused to participate in the study or inaccurately filled out the survey.

### Sampling

The survey was distributed in Arabic and English from December 2023 to January 2024. We announced on social media that we were seeking national leaders to assist in distributing the survey within their respective regions. Interested individuals signed up through social media links, and we communicated with them to provide clear instructions on how to distribute the survey. Each national leader was given a unique link to the form and asked to either distribute it digitally or print and share it within their universities. National leaders were selected based on their willingness to participate and their affiliation with key student organizations or academic institutions, ensuring diverse regional coverage. No financial incentives were offered; instead, leaders participated voluntarily to contribute to the research effort.

We utilized multiple social media platforms and collaborated with student organizations to recruit participants. The sampling process involved voluntary consent from students, who completed the surveys either online or through printed forms.

Regarding the sample size, calculations were performed to ensure adequate power. However, we did not make any specific adjustments for potential dropouts or incomplete responses, as the survey format was designed to minimize these risks by allowing participants to complete it at their convenience. PQStat Software (2021) v.1.8.2.238 (PQStat Software, Poznan, Poland) was used to calculate the sample size. The sample size was calculated according to the specific country setting for this multinational study. Assuming there are 1.9 million students in Jordan and 25 million in Egypt, with 95% confidence intervals, 5% margin of error, and a standard deviation of 0.5. The estimated minimum sample size was 384 for each country.

### Study tool

We divided our survey into two three sections:

1. consent to participate;
2. the demographic characteristics [age, gender, citizenship, frequency of watching social media posts on Gazza and Isreal war, having a relationship with a Palestinian individual (colleague, friend, neighbor, or relative), student status];
3. Brief Symptom Inventory 18 (BSI 18), which has three dimensions: somatization, depression, and anxiety. Each has six questions, and their answers are (extremely, quite a bit, moderately, a little bit, and not at all), scored from 4 to 0, respectively [17]. The sum of all scores indicates the General distress score, which is classified as 1) No elevation (t-scores < 50; 2), Minor elevation (t-scores between 50 and 56; 3), Moderate elevation (t-scores between 57 and 62; and 4) and Marked elevation (t-scores ≥ 63) [17]. The scales' internal consistency reliability was determined with Cronbach's standardized alpha [18].

### Ethical consideration

We conducted this study in accordance with the Declaration of Helsinki [19], and written informed consent was obtained from all participants. Ethical approval was not required because the study was classified as population surveillance rather than a clinical trial, focusing on attitudes rather than clinical interventions. The Bioethics Committee of Deraya University, Faculty of Pharmacy, issued an ethical waiver in accordance with the guidelines of the Council for International Organizations of Medical Sciences (CIOMS), the World Health Organization (WHO), and the Egyptian Clinical Trial Law (April 2018).

Despite the waiver, we ensured that participants' rights were fully protected. All participants were informed of the study’s purpose, their right to withdraw at any time, and the confidentiality of their responses. Data were anonymized and securely stored, ensuring compliance with all relevant data protection standards.

### Statistical analysis

We performed the statistical analysis using PQStat Software (2021) v.1.8.2.238 (PQStat Software, Poznan, Poland). Listwise deletion was used for missing data. Shapiro–Wilk test was used to measure the normality of continuous data. Categorical data were reported as frequency/percentage and continuous data as mean/standard deviation (SD) (for normal distribution) or median/interquartile range (IQR) (for non-normal distribution). Mann–Whitney U test measured the differences in general distress, somatization, depression, and anxiety between females and males, friends, and country. Kruskal–Wallis ANOVA tested the scores of general distress, somatization, depression, anxiety, and frequency of watching social media posts. Moreover, Spearman's rank correlation coefficient (r) was used to investigate the correlation between age and general distress, somatization, depression, and anxiety. Finally, we built univariate and multi-logistic regression models for the elevated general distress (≥ 50) and combined moderate and marked general distress (≥ 57). The results were presented as odds ratios (ORs) and 95% confidence intervals (95% CI). A *P*-value less than 0.05 was statistically significant in all tests.

## Results

### Demographic characteristics of the participants

A total of 1509 participants were included in the analysis, of whom 66% were female. The average age was 22.75 ± 2.33. Most participants were watching social posts about war multiple times a day. Approximately 72% of the participants were Egyptian, and 28 were Jordanian. Also, around 61% had no Palestinian (friend, neighbor, colleague, or relative). Most of the students, 82%, show no elevation in the BSI-18 score (Table 1).

**Table 1:** Demographic data of the participants, frequencies reported as N (%)

**Age**	22.75 ± 2.33; [22 (20—23)]^*^
**Gender**	
Female	996 (66)
Male	513 (34)
**Nationality**	
Egypt	1081 (71.6)
Jordan	428 (28.4)
**Follow social media posts about the Israel-Gaza conflict in the past two months**	
Multiple times a day	1176 (77.9)
Once a day	185 (12.3
A few times a week	110 (7.3)
Once a week	15 (1)
Rarely	20 (1.3)
Never	3 (0.2)
**Do you have a Palestinian (friend, neighbor, colleague, or relative)?**	
No	917 (60.8)
Yes	592 (39.2)
**BSI-18 total score**	
No elevation	1240 (82.2)
Minor elevation	121 (8)
Moderate elevation	56 (3.7)
Marked elevation	92 (6)

^*^reported as mean ± SD; [median (IQR)]*

### BSI-18 questionnaire scoring

For all participants, the general distress score was 30.33 ± 18.7. the lowest score was for the somatization domain, with 7.56 ± 6.3, followed by anxiety and depression, with 10.76 ± 7.4 and 12.02 ± 6.9, respectively. The scales' Cronbach's standardized alpha demonstrated an excellent reliability of α = 0.0.956 [18]. The mean and standard deviation of BSI-18 domains, along with each item correctional and its Cronbach Alpha, are shown in Table 2.

**Table 2:** Scores for domains of somatization, depression and anxiety, and general distress score

Domain	Mean (standard deviation)	Item-total scale correlation	Cronbach Alpha, if the item is deleted
**Somatization**	7.56 (6.3)		
Faintness or dizziness	0.82 (1.1)	0.59	0.96
Pain on heart or chest	1.39 (1.3)	0.66	0.95
Nausea or upset stomach	1.42 (1.3)	0.68	0.95
Trouble getting one's breath	1.30 (1.3)	0.71	0.95
Numbness or tingling in parts of one's body	1.15 (1.3)	0.67	0.95
Feeling week in part of one's body	1.49 (1.3)	0.72	0.95
**Depression**	12.02 (6.9)		
Feeling no interest in things	2.23 (1.4)	0.71	0.95
Feeling lonely	2.06 (1.4)	0.77	0.95
Feeling blue	2.51 (1.3)	0.74	0.95
Feeling worthless	2.16 (1.5)	0.72	0.95
Feeling hopeless about the future	2.28 (1.5)	0.72	0.95
Thoughtful of ending one's life	0.78 (1.3)	0.58	0.96
**Anxiety**	10.76 (7.4)		
Nervosenous of shaking inside	1.89 (1.4)	0.79	0.95
Feeling tense or keyed up	2.16 (1.4)	0.80	0.95
Feeling so restless that one could not sit still	1.69 (1.4)	0.80	0.95
Suddenly scared for no reason	1.84 (1.5)	0.81	0.95
Spells of terror or panic	1.25 (1.4)	0.74	0.95
Feeling fearful	1.93 (1.5)	0.78	0.95
**General distress**	**30.33 (18.7)**		

### Differences between gender and age and distress

**Fig. 1:**
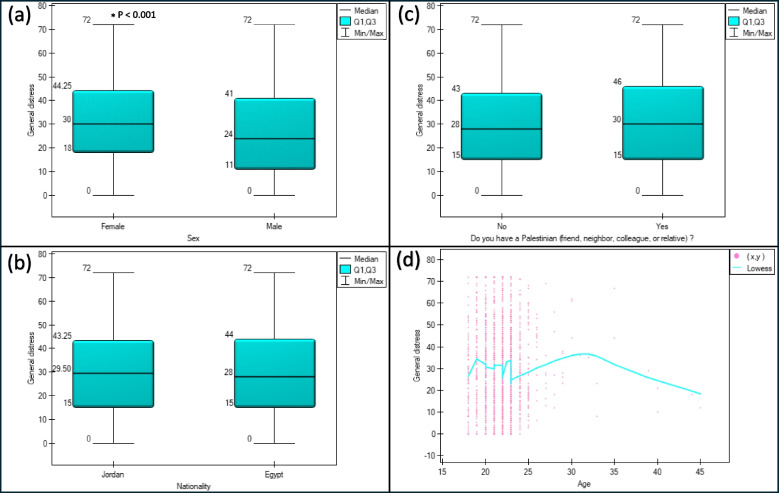
Differences in general distress score according to (**a**) gender, (**b**) nationality, (**c**) relationship with a Palestinian, and (**d**) Spearman's rank correlation between general distress and age

Females scored significantly higher in general distress 30 (18–44.25) vs. 24 (11–41), *p* < 0.001 (Fig. 1a) and all three dimensions (Table 3). Moreover, we did not find any significant correlation between age and any of the dimensions (R somatization = -0.015, *p* = 0.56), (R depression = 0.017, *p* = 0.506), (R anxiety = -0.015, *p* = 0.553) or the total score (R general distress = -0.006, *p* = 0.822) (Fig. 1d).

**Table 3:** The influence of demographic differences on the general distress, somatizaiton, depression, and anxiety scores

	**General distress**	**Somatization**	**Depression**	**Anxiety**
**Gender**				
Male	24 (11–41)	4 (1–11)	11 (5–18)	8 (2–15)
Female	30 (18–44.25)	7 (3–12)	12 (7–18)	11 (6–18)
*p*-value	< 0.001	< 0.001	0.002	< 0.001
**Having a Palestinian (friend, neighbor, colleague, or relative)**				
Palastinan relationship	30 (15–46)	6 (3–12)	12 (6–18)	11 (4–18)
Palastinan relationship	28 (15–43)	6 (2–11)	12 (6–18)	10 (4–17)
*p*-value	0.094	0.047	0.249	0.132
**Country**				
Egypt	28 (15–44)	6 (3–12)	12 (6–18)	10 (5–17)
Jordan	29.5 (15–43.25)	5 (2–10)	13 (7–18)	11 (4–18)
*p*-value	0.941	< 0.001	0.005	0.85
**Frequency of following social media posts about the Israel-Gaza conflict in the past two months**				
Multiple times a day	30 (16–46)^a, b^	6 (3–12) ^a, b, c^	12 (6–18)^a, b^	11 (5–18)^a, b^
Once a day	27 (16–38)^c^	5 (2–9)^c^	12 (7–17)^c^	9 (5–15)^c^
A few times a week	20 (11–32.5)^b^	5 (1–8)^b^	8 (5–14.75)^b^	6 (3–11.75)^b, c^
Once a week	18 (13–27)	5 (1.5–7)	7 (5.5–10)	7 (3–8)
Rarely	13 (5.75–22)^a, c^	2 (0–5)^a^	5 (2–9)^a, c^	6.5 (1–9.25)^a^
Never	16 (11–21)	4 (4–6)	2 (2–6)	8 (4–9)
*p*-value	< 0.0001	< 0.0001	< 0.0001	< 0.0001

^a^, ^b^, and ^c^ are denoted for the statistical significance of the relationship between values with the same letter. For example, in somatization domain, "multiple times a day" have a significant relationship with "once a day", "a few times a week" and "rarely"

### Differences between countries and having a relationship with a Palestinian

Egypt had a higher somatization score than Jordan 6 (3–12) vs 5 (2–10), *p* < 0.001. Jordan had higher depression than Egypt 13 (7–18) vs. 12 (6–18), *p* = 0.005. no significant differences were observed in General distress or Anxiety (Table 3, Fig. 1c).

Moreover, somatization was higher in those having a relationship with a Palestinian 6 (3–12) vs. 6 (2–11), *p* = 0.047. No other significant differences were found in depression, anxiety, or total general distress score (Table 3, Fig. 1b).

### Influence of watching social media posts

Overall, there were significant differences between the frequency of watching social media posts and distress. Post-hoc analysis showed that watching social media posts multiple times a day was significantly higher in general distress than rarely 30 (16–46) vs. 13 (5.75–22), *p* < 0.001 (Table 3). Post-hoc analysis for anxiety, depression, and somatization revealed that watching social media posts multiple times a day was also significantly higher in general distress than rarely. Few observations were also noted between once a day, rarely, and even a few times a week. Full results are shown in (Table 3).

### General distress predictors

The univariate predictors for elevated general distress (≥ 50) were being Egyptian (OR 1.49, 95% CI 1.08 to 2.08, *p* = 0.014), watching social media multiple times a day (OR 3.04, 95% CI 1.966 to 4.688, *p* < 0.001), and having a Palestinian connection (friend, neighbor, colleague, or relative) (OR 1.63, 95% CI 1.232 to 2.161, *p* < 0.001). These three predictors were retained in the backward stepwise multivariate regression analysis model (Table 4).

**Table 4:** Univariate and multivariate models for the elevated general distress (≥ 50)

Predictor	b coeff	b error	Wald stat	*p*-value	odds ratio	-95% CI	+ 95% CI
Univariate predictors							
Age	-0.048	0.032	2.286	0.131	0.953	0.895	1.014
Sex	-0.234	0.148	2.490	0.115	0.792	0.592	1.058
Nationality	0.405	0.166	5.989	0.014	1.500	1.084	2.075
Watch social media multiple times a day	1.111	0.222	25.108	< 0.001	3.036	1.966	4.688
Having a Palestinian (friend, neighbor, colleague, or relative)	0.490	0.143	11.667	0.001	1.632	1.232	2.161
Backward stepwise multivariate regression analysis							
Nationality	0.376	0.163	5.318	0.021	1.457	1.058	2.006
Watch social media multiple times a day	1.102	0.221	24.771	< 0.001	3.009	1.950	4.643
Having a Palestinian (friend, neighbor, colleague, or relative)	0.460	0.142	10.474	0.001	1.585	1.199	2.094

Meanwhile, the univariate predictors for moderate and marked general distress (≥ 57) were watching social media multiple times a day (OR 3.26, 95% CI 1.780 to 5.987, *p* < 0.001), and having a Palestinian connection (friend, neighbor, colleague, or relative) (OR 1.4993, 95% CI 1.049 to 2.142, *p* = 0.026). The backward stepwise multivariate regression analysis model retained only the predictor of watching social media multiple times daily (Table 5).

**Table 5:** Univariate and multivariate models for the for moderate and marked general distress (≥ 57)

**Predictor**	b coeff	b error	Wald stat	*p*-value	odds ratio	-95% CI	+ 95% CI
**Univariate predictors**							
Age	-0.027	0.040	0.476	0.490	0.973	0.900	1.052
Sex	0.046	0.184	0.063	0.801	1.047	0.731	1.501
Nationality	0.394	0.215	3.350	0.067	1.483	0.972	2.261
Watch social media multiple times a day	1.183	0.309	14.626	< 0.001	3.265	1.780	5.987
Having a Palestinian (friend, neighbor, colleague, or relative)	0.405	0.182	4.947	0.026	1.499	1.049	2.142
Backward stepwise multivariate regression analysis							
Watch social media multiple times a day	1.252	0.308	16.547	< 0.001	3.498	1.913	6.395

## Discussion

We found that 17.8% of the students had an elevation in their BSI-18 total score. Females experienced higher general distress than males. Individuals who watched daily social media posts related to war exhibited significantly higher general distress than those who rarely watched such content. The frequency of social media watching and distress was also considered. Additionally, no significant correlation between age and general distress was observed. Egyptian and Jordanian students, regardless of whether they had a Palestinian connection, had comparable general distress scores. Multivariate predictors for elevated general distress (≥ 50) were being Egyptian, watching social media multiple times a day, and having a Palestinian connection. Meanwhile, the primary predictor for moderate and marked general distress (≥ 57) was watching social media multiple times daily.

### Gender and age

We found that females reported higher general distress, somatization, depression, and anxiety (Table 3). This agrees with previous findings on students [10, 20–24] and university teachers [25]. Furthermore, a systematic review by Xiong et al. [26] encompassing 19 studies and a total of 93,569 participants found that females were significantly more likely to experience mental distress compared to males [26].

As for age, no noticeable differences were observed in our analysis, given that the students were within the same age range. Al-Tammemi et al. reported different results, as being older was a protective factor against higher levels of distress (adjusted OR = 0.64, 95% CI 0.44 to 0.94, *p* = 0.022). Similarly, Saravanan et al. reported that higher age was 13% less likely to have psychological Distress (OR 0.87, 95% CI 0.80 to 0.95, *p* = 0.002) [27]. Sansgiry and Sail highlight that Student age was significantly correlated with (r = 0.91, *p* < 0.001) anxiety – younger students reported lower test anxiety than older students [28].

### War, students and psychological distress

We found that 17.8% of the students had an elevation in their BSI-18 total score. However, these students were not considered inside the war zone, albeit in countries with borders with either Gazza, Egypt, or Palestine, generally, Jordan. This situation was similar to the Hisato et al. study, where students located in Poland, which shares a border with Ukraine, showed that anxiety, stress, and depression were significantly associated with political instability in Eastern Europe due to the war between Ukraine and Russia) (r = 0.178, r = 0.169, and r = 0.154, respectively) [23]. Mollica et al. highlighted that war can have a profound impact on adolescents. During such conflicts, they are exposed to horrific events, organized violence, the breakdown of social networks, and displacement. These experiences occur during critical physical, emotional, social, and cognitive development stages [29]. Consequently, these experiences pose severe risks to their physical health and psychological development [30]. Several observations were noted as well about how war influences psychological distress in students [13, 14, 23, 31–36] (Table 6).

**Table 6:** Signs of psychological distress for students worldwide due to war

Country of the study	Country/region in conflict	Number of affected Students	Total number of students	Percentage of students with psychological distress	Observation time		
					from	To	Reference
**Syria**	Syria	212	350	60.6	Nov 2015	Mar 2016	[14]
**Poland**	Ukraine	196	461	42.5	Mar 2022	April 2022	[23]
**Uganda**	North of Uganda	117	205	57	June 2010	Jul 2010	[32]
**Libya**	Civil war	1896	2430	78	April 2020	May 2020	[13]
**Yemen**	Yemen	631	1051	60	Nov 2016	Jul 2017	[33]
**Palestine**	Gaza	399	399	100	Nov 2012	Jul 2013	[34]
**Czech**	Ukraine	405	591	69	Mar 2022	May 2022	[35]
**Bosnia and Herzegovina**	Bosnia and Herzegovina	34	78	44	June 2004	Jul 2004	[36]
**Ukraine**	Ukraine	576	589	97.8	May 2022	May 2022	[31]

### Social media news

What was more alarming was that daily watch of social media posts was not only higher than those rarely watching (Table 4), but it was a predictor for both elevated general distress (≥ 50) and for moderate and marked general distress (≥ 57). Research studies have shown that being exposed to news stories about war can hurt people's emotional health. However, there has not been research into how daily exposure to news affects individuals differently. It is essential to delve into the downsides of consuming news regularly and understand when these effects might appear [37]. During the Ukrainian-Russian wartime, participants started feeling better as the conflict became less prominent in the media. However, this improvement was influenced by their personality traits [38]. It is suggested that these set points can shift under the circumstances, showing that people adapt differently to life's difficulties and that there is a link between people's well-being and the war-related content they see in media [38]. Days, with many war-related posts were linked to reported levels of well-being, showing how media exposure can affect our emotions [38].

A study conducted on 591 students in the Czech Republic, of which 63.8% were medical students, found that many were already following the news about the Russian-Ukrainian war. The study revealed that 34% of the participants expressed anxiety, and 40.7% reported suffering from depression due to the instability in Europe [35]. In another study conducted in Italy with over 520 participants, the researchers aimed to study the aggressive reactions associated with the Russian-Ukrainian war and evaluate the effect of emotional cognition, including conflict-related emotions such as anger and shame [39]. The results indicated a significant correlation between aggressive reactions and negative emotions like anger and shame. The mean scores (and standard deviations) for aggressive reactions, anger, and shame were 2.28 (± 0.85), 3.04 (± 1.28), and 2.33 (± 1.46), respectively [39].

### Relationship with Palestinians

We found that having a Palestinian (friend, neighbor, colleague, or relative) resulted only in having a somatization score. Still, this factor was a common univariate predictor for elevated and moderate to marked general distress. Having a friend whose country is at war can significantly impact a student's general distress. Students may experience future anxiety, which can be exacerbated by the uncertainty and instability associated with war [40]. This anxiety can be particularly pronounced among international students who come from areas experiencing war and conflict [40]. The social fabric of their lives can be disrupted, leading to a sense of insecurity and fear [41]. Lessons from Myanmar showed that these experiences can lead to a constellation of social and economic problems rather than just a list of mental health symptoms [42]. It is also important to highlight that students with worse relationships with family and friends were at a higher risk of social isolation and mental health problems [43]. Therefore, academic institutions play a crucial role in reducing psychological distress among students. They can provide support through various mechanisms, such as mindfulness, decentering, reappraisal, and emotion regulation [44]. These strategies can help students manage stress and anxiety, which are common among university students [45]. Furthermore, academic institutions can create a sense of security and stability for students, which is particularly important for those dealing with external stressors such as war or conflict [45]. By fostering a supportive and understanding environment, academic institutions can help students navigate the challenges of university life and reduce psychological distress [44, 45].

## Strength and limitations

We conducted a cross-sectional study that reached the estimated sample size and showed an elevated distress level associated with war in Gazza. To our knowledge, it is the first study assessing the psychological distress among students in Egypt and Jordan after only two months of the war. We used robust scale and strict inclusion and filtration criteria to allow proper statistical power. For example, even though the study is intended to be filled out by students only, we have added a demographic question asking about student status because if the survey is distributed online, there will be a chance that anybody can fill it out. Hence, we were able to distinguish who filled out the survey accurately. Also, the average time to complete the survey was 2 min and 23 s; therefore, we intended to filter out any suspicious response that could have been filled with a bot. Fortunately, after cleaning the data, responses had higher integrity. However, we wish to stress some of the limitations of our study. First, the survey was distributed onsite and online; however, access to digital platforms may have posed a barrier for some participants, particularly those with limited internet access or lower digital literacy, potentially affecting the representativeness of the sample. The study is based on self-reporting. Therefore, the declarations could not be confirmed on the objective ground; the perspective of pain might differ from one person to another. Also, the survey was distributed in Arabic and English to minimize the risk of unclarity. Although the current findings shed light on the impact on students from Egypt and Jordan, these results are subject to potential sampling biases. This is because the sample was opportunistic rather than a representative random sampling. Therefore, there may be limitations in generalizing these experiences to students living in other countries across the MENA regions. It will be interesting to see more countries, especially those in the MENA region, that do not share borders with Palestine. We did not measure other demographic factors such as socioeconomic status, grades, or academic discipline, which could have provided more insights into the data; still, we have focused our survey on one central theme to make the survey swift to fill and prevent the distraction of respondents. Finally, we acknowledge that the BSI-18 is a screening instrument and not a diagnostic tool. While it provides valuable insights into the prevalence of general distress, we are cautious in using terms like 'depression' and 'anxiety,' as these cannot be definitively diagnosed without thorough psychiatric evaluation. Therefore, we view the BSI-18 results as an initial assessment that signals the need for further clinical evaluation where high levels of distress are identified.

## Conclusion

Our results demonstrated a general increase in distress among students in Egypt and Jordan due to the War in Gaza. Females exhibited higher levels of general distress, anxiety, somatization, and depression than males. Students from Egypt who have connections with Palestinian individuals or who view social media posts about the war multiple times daily were at a higher risk of experiencing increased general distress. The latter were at a higher risk for moderate to marked elevation in general distress. Further research is required to address students' coping skills and identify other factors associated with anxiety, stress, and depression, such as socioeconomic status, grades, and academic discipline. It is strongly recommended that universities establish, maintain, and enhance mental health support programs accessible to all students.

## Supplementary Information

Supplementary Material 1.

## Data Availability

The datasets used and/or analyzed during the current study are available from the corresponding author upon reasonable request.
